# Potentiation of Electrochemotherapy by Anti-PD-1 Immunotherapy in Murine Tumors with Distinct Immune Profiles

**DOI:** 10.3390/cancers18010090

**Published:** 2025-12-27

**Authors:** Masa Omerzel, Simona Kranjc Brezar, Ursa Lampreht Tratar, Tanja Jesenko, Barbara Lisec, Gregor Sersa, Maja Cemazar

**Affiliations:** 1 Department of Experimental Oncology, Institute of Oncology Ljubljana, 1000 Ljubljana, Slovenia; momerzel@onko-i.si (M.O.); skranjc@onko-i.si (S.K.B.); ulampreht@onko-i.si (U.L.T.); tjesenko@onko-i.si (T.J.); blisec@onko-i.si (B.L.); gsersa@onko-i.si (G.S.); 2 Faculty of Health Sciences, University of Ljubljana, 1000 Ljubljana, Slovenia; 3 Faculty of Medicine, University of Ljubljana, 1000 Ljubljana, Slovenia; 4 Veterinary Faculty, University of Ljubljana, 1000 Ljubljana, Slovenia; 5 Faculty of Health Sciences, University of Primorska, 6310 Izola, Slovenia

**Keywords:** electrochemotherapy, bleomycin, immunotherapy, anti-PD-1, tumor immune status, murine models

## Abstract

**Background**: Electrochemotherapy (ECT) is a clinically validated local ablative treatment increasingly recognized for its ability to induce immunogenic cell death and stimulate antitumor immunity. Its combination with immune checkpoint inhibitors, such as anti-PD-1 antibodies, may enhance systemic immune responses and improve therapeutic efficacy, particularly in poorly immunogenic tumors. **Methods**: We evaluated the antitumor effectiveness of ECT combined with a murine analog of the anti-PD-1 antibody in four syngeneic murine tumor models with differing histology and immune status: WEHI fibrosarcoma, CT26 and MC38 colorectal carcinoma, and 4T1 mammary carcinoma. In vitro cytotoxicity assays assessed tumor cell sensitivity to ECT, while in vivo experiments evaluated complete response (CR) rates, immune cell infiltration, and long-term immune memory through secondary tumor challenge. Immunohistochemical analysis of CD4^+^, CD8^+^, and granzyme B^+^ effector cells. **Results**: In vitro, WEHI cells exhibited the highest sensitivity to ECT. In vivo, ECT monotherapy induced CRs in 100% of WEHI tumors, 60% of CT26, 17% of 4T1, and 15% of MC38. The addition of anti-PD-1 significantly enhanced outcomes in less responsive models, increasing CRs to 90% in CT26, 91% in MC38, and 53% in 4T1. Combination therapy promoted pronounced infiltration of CD4^+^, CD8^+^, and granzyme B^+^ T cells and the formation of tertiary lymphoid structure, particularly in MC38 tumors. Secondary challenge experiments confirmed long-term immune memory in CT26 and MC38 models and induced memory in 4T1, which was absent following monotherapy. **Conclusions**: ECT synergizes with PD-1 blockade to potentiate local and systemic antitumor immunity, overcoming immune resistance in poorly immunogenic tumors. These findings support further clinical development of ECT in combination with immune checkpoint inhibitors as a component of personalized cancer immunotherapy.

## Simple Summary

Electrochemotherapy (ECT) is a well-established local ablative therapy that also exerts immunomodulatory effects, making it a promising combination partner for immunotherapy. This study evaluated the therapeutic efficacy of ECT combined with anti-PD-1 antibody treatment in several murine tumor models with distinct histology and immune profiles. The results demonstrate that ECT can elicit systemic immune activation and durable antitumor memory when combined with anti-PD-1 therapy, particularly in tumors with low intrinsic immunogenicity, supporting its potential role in personalized cancer immunotherapy.

## 1. Introduction

Electrochemotherapy (ECT) is a local cancer treatment that combines administration of a cytotoxic drug with electroporation to increase drug uptake. The application of electrical pulses transiently increases cell membrane permeability, allowing for increased intracellular concentrations of cytotoxic drugs such as bleomycin (BLM), cisplatin, and oxaliplatin, thereby promoting efficient tumor cell death [1,2]. ECT was initially developed for the treatment of various types of cutaneous and subcutaneous tumors, and more recently, deep-seated tumors, which have gained attention for their potential immunomodulatory effects [3,4,5,6,7,8]. In addition to its high local response rates and limited systemic toxicity, ECT has emerged as a promising inducer of immunogenic cell death (ICD) in specific tumor types. ICD stimulates the release of tumor-associated antigens and damage-associated molecular patterns (DAMPs), thereby activating an antitumor immune response [9,10].

In a study by Calvet et al., CT26 murine colon cancer cells treated with ECT BLM in vitro exhibited hallmarks of ICD, including release of DAMPs [9]. When these treated cells were injected into immunocompetent mice, they induced a robust immune response that prevented subsequent tumor growth. Similarly, Kesar et al. demonstrated that ECT with BLM, cisplatin, or oxaliplatin induced immunologically relevant changes in vitro, reinforcing the immunostimulatory potential of this therapy. However, we have to emphasize that listed cytotoxic drugs differ significantly in their capacity to induce ICD [11].

In some of the published studies ECT has been proposed as an in situ vaccination strategy capable of converting immunologically “cold” tumors into “hot” ones by enhancing antigen presentation and promoting T-cell infiltration [12,13,14]. Although ECT indeed increases antigen release and immune infiltration, current evidence does not demonstrate consistent conversion of immune-excluded tumors into fully inflamed phenotypes. Various preclinical and clinical studies have explored combined therapies to amplify the immunostimulatory effects of ECT [1,3].

Over the past decade, immunotherapy with immune checkpoint inhibitors (ICIs) has transformed cancer treatment, offering durable responses in several advanced-stage malignancies by reactivating the host immune system to target the cancer cells [15]. However, the effectiveness of immunotherapy varies across tumors with different histological and immunological profiles. While tumors, such as melanoma and non-small cell lung cancer, tend to respond well, immunologically “cold” tumors frequently show limited benefits [16].

A recent study by Campana et al. investigated the combination of ECT with the immune checkpoint inhibitor pembrolizumab in patients with advanced skin melanoma, revealing significantly improved local progression-free survival, systemic progression-free, and overall survival in the combined ECT-pembrolizumab group compared with those in the pembrolizumab monotherapy group [3]. This synergy was probable, since melanoma is intrinsically immunogenic tumor; however, it cannot be directly translated to tumors with low baseline immunogenicity.

To the best of our knowledge, ICI have not been tested using ECT in preclinical settings. However, other forms of immunotherapy, such as IL-12 gene electrotransfer (GET), have been successfully combined with ECT, confirming the rationale for such an approach [1]. In the present study, we aimed to investigate the interplay between ECT and immunotherapy using a murine analog of anti-PD-1, assess their combined effects on murine tumors, clinically relevant for ECT, with different histological and immunological profiles, and explore their potential to enhance antitumor immune responses.

## 2. Materials and Methods

### 2.1. Cell Lines

Four murine cell lines, MC-38, 4T1, WEHI 164 (WEHI), and CT26, were used in this study. Murine colorectal carcinoma MC38 cells (Kerafast, Newark, CA, USA) were cultured in Advanced DMEM (Gibco, Thermo Fisher Scientific, Waltham, MA, USA). Mammary carcinoma 4T1 cells, murine fibrosarcoma WEHI cells, and murine colorectal carcinoma CT26 cells (all from the American Type Culture Collection, Manassas, VA, USA) were cultured in Advanced RPMI-1640 medium (Gibco, Waltham, MA, USA). Both media were supplemented with 5% fetal bovine serum (FBS; Gibco), GlutaMAX (100X, Gibco), and Penicillin–streptomycin (100×, Sigma-Aldrich, Merck, Darmstadt, Germany). Although the cells were grown in different media, this may not influence the sensitivity of cells to ECT since the cells were exposed to ECT in HBSS. The cells were routinely tested for mycoplasma infection using a MycoAlertTM PLUS Mycoplasma Detection Kit (Lonza, Basel, Switzerland), and were mycoplasma-free.

### 2.2. Solutions

For in vitro experiments, BLM (15000 IU bleomycin, Bleomycin Medac, Medac, Germany) was diluted in HBSS (without calcium, magnesium, or phenol red; HBSS^−/−^; Gibco) to 1 µg/mL, 0.1 µg/mL, 0.01, 0.001, and 0.0001 µg/mL, respectively, in a final cell suspension. The wide concentration range (10^−4^–1 µg/mL) was selected to capture the broad spectrum of BLM susceptibility reported for murine cancer lines and to reliably calculate IC_50_ values under EP and non-EP conditions.

For in vivo experiments, BLM (Medac) was diluted in 0.9% NaCl saline (B. Braun Melsungen AG, 3632563, Melsungen, Germany) to a concentration of 3 mg/mL.

### 2.3. In Vitro Electroporation and Cytotoxicity Assay

The cells were trypsinized, collected by centrifugation, washed in ice-cold HBSS^−/−^, and prepared for electroporation (EP). Briefly, 20 µL of BLM (concentrations described in Section 2.2) was added to 80 µL of the cell suspension (2 × 10^6^ cells). One half (50 μL) was used as the treatment control, while the other half was pipetted between two custom-made stainless-steel parallel-plate electrodes (2 mm apart), and electric pulses were applied (eight square-wave pulses, 1300 V/cm, 100 μs, and 1 Hz) with an electric pulse generator GT-01 (Faculty of Electrical Engineering, University of Ljubljana, Ljubljana, Slovenia). The EP parameters were chosen based on clinically validated ESOPE pulse regimens and prior optimization studies showing efficient permeabilization with minimal mechanical damage. The cells were subsequently transferred to a 24-well ultra-low attachment plate and incubated for 5 min at room temperature, followed by the addition of 1 mL of culture medium. A viability assay using Presto Blue (Thermo Fisher Scientific) was performed to determine cell viability after treatment with BLM alone or in combination with EP. The cells were diluted to 1 × 10^4^ cells/mL (control groups) or 2 × 10^4^ cells/mL (EP groups), and 100 µL was transferred to 96-well plates, with each well containing 1 × 10^3^ cells (exposed to the drug alone) or 2 × 10^3^ cells (exposed to the combined treatment with EP). EP reduces immediate post-pulse viability; therefore, higher initial seeding ensured equivalent post-treatment cell numbers at assay start. Normalizing fluorescence to EP and non-EP controls avoided bias. The plates were incubated for 72 h in a humidified atmosphere of 5% CO_2_ at 37 °C. Presto Blue reagent (10 μL/well) was added to the cells, followed by a 1 h incubation in a humidified incubator at 5% CO_2_ and 37 °C. The fluorescence emission was measured using a Cytation 1 microplate reader (BioTek Instruments, Winooski, VT, USA) with a 530/590 nm excitation/emission filter. The measured fluorescence intensity of the treated groups was normalized to that of the control and EP groups.

### 2.4. Animals

Female C57Bl/6NCrl (C57Bl/6) mice were obtained from Charles River Laboratories RMS (Germany GmbH, Sulzfeld, Germany) and BALB/cOlaHsd mice were obtained from Envigo RMS Srl (San Pietro al Natisone, Udine, Italy). The mice were 8–10 weeks old and weighed 19–21 g. Six mice per cage were housed in a specific pathogen-free environment under controlled conditions in a carousel mouse IVC rack system (Animal Care Systems Inc., Centennial, CO, USA) with a 12 h light–dark cycle, a temperature range of 20–24 °C, and a relative humidity of 55 ± 10%. The mice were provided ad libitum access to a standard rodent diet (Altromin 1324, Altromin Spezialfutter GmbH & Co. KG, Lage, Germany) and water with corncob bedding (Rehofix MK 2000, J. Rettenmaier & Söhne GmbH + Co. KG, Rosenberg, Germany) was used as the substrate. Each cage was equipped with environmental enrichment items for rodents, including a paper e-cube (Allentown Deutschland GmbH, Neuss, Germany), wooden brick (TAPVEI Estonia Oü, Hurjumaa, Estonia), and red-sheltered tunnel (Animalab, Poznań, Poland). All procedures were conducted in accordance with the EU Directives for Animal Experiments and the ARRIVE Guidelines, and were approved by the Ministry of Agriculture, Forestry, and Food of the Republic of Slovenia (permission no. U34401-35/2020/8 and U34401-3/2022/17, 18 September 2024). During the BLM ECT (ECT) procedure, the mice were anesthetized with 2% (*v*/*v*) isoflurane (Isoflurane; Piramal Healthcare UK Limited, London, UK). All experiments were designed and performed according to the 3R principle and the ARRIVE, PREPARE, and OBSERVE guidelines [17,18,19]. Mice were randomly assigned to experimental groups, as suggested by Labcat Animal Study Software, version 8.0.102, each consisting of 15–20 animals, with the exact number specified in the corresponding graphs and figure captions. Sample size was calculated using the statistical power analysis software G*Power 3.1.9.7. We applied the test for calculating the sample size required to detect differences between proportions in two independent groups using Fisher’s exact test. We assumed a difference between proportions of 0.6, an α level of 0.05, a test power of 0.95.

The general health and body weight of the animals were monitored daily, with a health assessment based on the coat condition and overall demeanor.

### 2.5. Tumor Induction and Treatment

For tumor induction, a suspension of 3 × 10^5^ MC38, 3 × 10^5^ 4T1, 2 × 10^6^ WEHI, or 3 × 10^5^ CT26 cells in 100 µL of NaCl was injected subcutaneously into the right flanks of syngeneic mice as previously described in the literature. When the tumors reached a volume of 45 mm^3^ (±5 mm^3^), the mice were randomly assigned to experimental groups. Tumor growth was monitored throughout the study period. Tumor types were selected based on relevancy to clinical practice. Melanoma, breast carcinoma, sarcomas and (liver metastases of) colorectal carcinoma are commonly treated with ECT, while anti-PD-1 is a standard therapy or investigated in clinical studies for listed tumor types.

**Figure 1:**
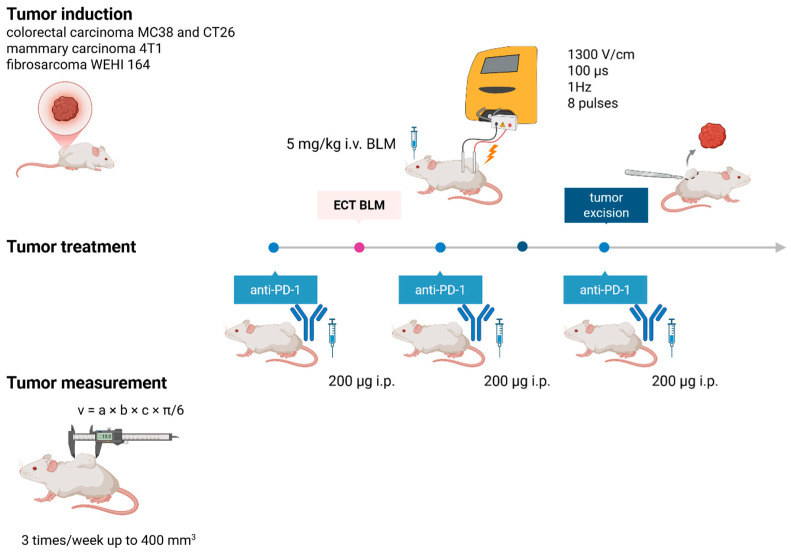
Study design. Schematic representation of tumor induction treatment and measurement. Created in BioRender. Cemayar, M. (2025) https://BioRender.com/1rkal6w (accessed on 26 August 2025).

In the combination therapy group, a murine analog of anti-PD-1 was administered every two days (three times in total) (Figure 1). For PD-1 blockade, InVivoMab anti-mouse PD-1 (CD276) (clone: [RMP1-14], Rat, Monoclonal, BIOZOL BXC-BE0146-100MG. Lot 861023M2) was used at a concentration of 10.22 mg/mL. The RMP1-14 binds to the extracellular domain of murine PD-1 with high affinity and effectively blocks the interaction of PD-1 with both of its ligands. Its rat IgG2a isotype has minimal Fc-mediated effector functions, preventing depletion of PD-1–expressing T cells and ensuring the checkpoint blockade. The antibody was administered intraperitoneally (i.p.) after dilution to a final concentration of 1 mg/mL (200 µg/200 µL) in physiological saline. One day after the first administration of anti-PD-1, an ECT of the subcutaneous tumors was performed. Bleomycin was administered via an intraorbital sinus injection, since it enables consistent systemic exposure and rapid delivery, at a dose of 5 mg/kg [20]. Each animal was weighed, and an appropriate volume of bleomycin was injected based on body weight. Three minutes after intravenous bleomycin administration, electrical pulses were generated via an electric pulse generator (ELECTRO cell B10, Betatech, Saint-Orens-de-Gameville, France) and applied in two perpendicular sets of four pulses via parallel stainless-steel electrodes under the following parameters: 1300 V/cm, 100 µs pulse duration, and 1 Hz frequency [20]. Non-invasive, plate electrodes (two parallel electrodes with a 6 mm gap) were used for the procedure. Ultrasound conductive gel (Ultraschall Gel, P.J. Dahlhausen, & Co. GmbH, Koln, Germany) was used to ensure good contact between the electrodes and the skin. The combined drug regimen was based on drugs pharmacokinetics and tried to simulate clinical settings.

The experimental groups were as follows: an injection of saline solution (control) combined with electroporation (EP); an injection of BLM, either alone (BLM) or in combination with EP (ECT); an anti-PD-1 agent, both as a standalone treatment (anti-PD-1) and in conjunction with EP (EP + anti-PD-1); a BLM combined with anti-PD-1 without EP (BLM + anti-PD-1); and finally, an anti-PD-1 agent combined with ECT (ECT + anti-PD-1).

The primary endpoint was treatment effectiveness, which was, measured by tumor diameters (a × b × c × π/6). If tumor failed to respond completely (CR), growth delay (GD) was measured as described in Section 2.6. Tumor GD was determined as the difference in the doubling time of tumors between the control and treated groups. Doubling time was calculated from growth curves. Partial responders, which eventually relapse, were included in the growth curve measurement and analysis.

### 2.6. Tumor Growth Measurement

Following treatment, tumor growth was monitored three time per week using a caliper (Model CD15DAX, Mitutoyo, Kanagawa, Japan). The measurements were recorded using the In-Life&Tumor software, version 8.0.102 (Labcat, Innovative Programming Associates, Inc., Princeton, NJ, USA) using the ellipsoid volume formula ((a × b × c × π/6), where a, b, and c represent perpendicular tumor diameters). Investigators were not blinded during tumor measurement. Euthanasia was performed when the primary or secondary tumor reached a predetermined humane endpoint of 400 mm^3^.

Tumor growth delay (GD) was determined as the difference in the doubling time of tumors between the control and treated groups. Animal weight loss and behavior were assessed using a mouse grip scale at every tumor measurement, as a sign of systemic toxicity of the treatments.

Mice that exhibited a complete response (CR, tumor-free for 100 days) were subjected to a secondary challenge with subcutaneous injection, of 3 × 10^7^ MC38, 3 × 10^7^ 4T1, 2 × 10^6^ WEHI, or 3 × 10^7^ CT26 tumor cells in 100 µL of NaCl to the opposite (left) flank that primary tumor was implanted. Tumor outgrowth was monitored, and if tumors failed to develop, mice were observed for an additional 100 days.

### 2.7. Histological Analysis and Immunohistochemistry

Tumors were excised on day 3 after treatment, fixed overnight in formalin (BD Pharmingen, BD Biosciences, San Jose, CA, USA), and subsequently embedded in paraffin. Consecutive 2-µm thick sections were cut from each paraffin block for histological and immunohistochemical analysis. The first section was stained with hematoxylin and eosin (H&E) to assess the percentage of necrotic tumor area. The second section was stained to evaluate the immunological effects of the granzyme B (cytotoxic effector cells), CD4 (helper T cells), and CD8+ (cytotoxic T cells) antibodies. Before staining with the primary antibodies, antigen retrieval was performed in sodium citrate buffer (10 mM sodium citrate, pH 6.0) at 95 °C for 30 min. The tumor sections were then covered with granzyme B (1:1000 dilution, ab4059, Abcam, Cambridge, UK), CD4 (1:100 dilution, ab183685, Abcam), or CD8+ (1:200 dilution, ab209775, Abcam) antibodies at 4 °C in a humid chamber. For detection, a rabbit-specific HRP/DAB (ABC) detection IHC kit (ab64261, Abcam) was used, following the manufacturer’s protocol. Nuclear counterstaining was performed using hematoxylin. The immunostained slides were scanned using a Nanozoomer slide scanner (Hamamatsu Photonics, Shizuoka, Japan), and the images were analyzed using the NDP.view2 viewing software, version 2.7.25 (Hamamatsu Photonics). Tumor necrosis and immune cell infiltration were assessed by examining the entire tumor slide and were quantified as percentages. Additionally, the number of granzyme B-, CD4-, and CD8-positive cells was analyzed and reported as the number of positive cells per 10 high-power fields (HPFs).

To evaluate the immunological effects collectively, an immune-associated score was calculated from the analyzed images. The number of CD4+, CD8+, and granzyme B-positive cells in untreated control tumors per 10 HPFs and lymphocyte infiltration served as a baseline for score determination. A higher number/10 HPFs were given a score of one point, and lower numbers were given proportionally fewer points (0.5 or 0.25). All other samples, after ECT or ECT + anti-PD-1 treatment, were than analyzed/counted to obtain number of cells/10 HPFs. The numbers were normalized to the baseline score, and scored proportionally higher or lower. HPFs were randomly assigned by technical associate not involved in image analysis. Positive cells were counted manually.

### 2.8. Statistical Analysis

Unless otherwise stated, all values in this study are presented as arithmetic mean (AM) ± standard error of the mean (SE). The normality of the data distribution was assessed using the Shapiro–Wilk test. Comparisons between two groups were conducted using an unpaired two-tailed Student’s *t*-test, whereas multiple group comparisons were analyzed using one-way analysis of variance (ANOVA), followed by Tukey’s multiple comparisons test. Statistical significance was set at *p* < 0.05. IC50 values were calculated using nonlinear regression analysis. Statistical analyses and graphical representations were performed using the GraphPad Prism software, version 10.6.1 (GraphPad, San Diego, CA, USA). Fisher’s exact test was as well performed using the GraphPad Prism software.

## 3. Results

### 3.1. Intrinsic Sensitivity of Tumor Cells to ECT In Vitro

**Figure 2:**
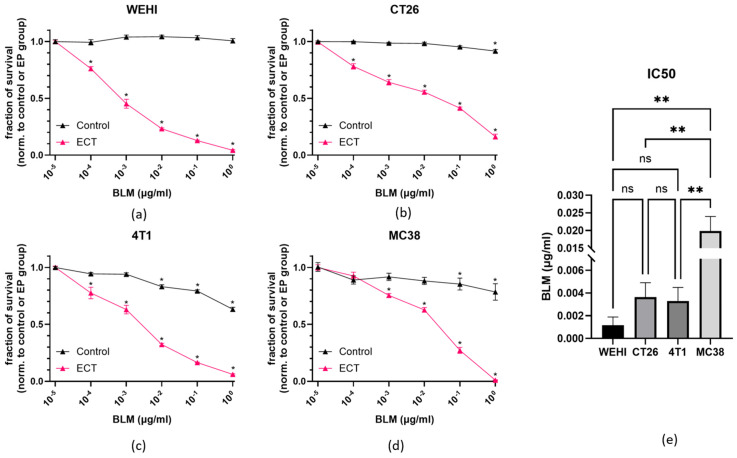
Intrinsic sensitivity of tumor cells to ECT in vitro. Intrinsic sensitivity of WEHI fibrosarcoma (**a**), CT26 colorectal carcinoma (**b**), 4T1 mammary carcinoma (**c**) and MC38 colorectal carcinoma (**d**) with corresponding IC50 values (**e**). * *p* < 0.05 indicates a significant difference compared to untreated control or EP group (**a**–**d**). ** *p* < 0.05 indicates a significant difference between indicated cell lines (**e**).

To evaluate the intrinsic sensitivity of different cancer cell lines to ECT, we assessed cell viability following treatment using the Presto Blue metabolic test (Figure 2). When BLM was administered alone, a small but significant reduction in cell survival was observed only at higher concentrations of bleomycin. However, when combined with EP, cell viability was significantly decreased in all cases, indicating a strong cytotoxic effect (Figure 2a–d). The half-maximal inhibitory concentration (IC50) values varied among cell lines, reflecting differential sensitivity to treatment. The IC50 values were 0.0012 µg/mL for WEHI, 0.0036 µg/mL for CT26, 0.0033 µg/mL 4T1 and 0.013 µg/mL for MC38 (Figure 2e). These results demonstrated that WEHIs were the most sensitive to ECT BLM, whereas MC38 exhibited the highest resistance among the tested cell lines. To better distinguish cytostatic from cytotoxic effects additional apoptosis-based or clonogenic assays would strengthen mechanistic interpretation.

### 3.2. Effectiveness of ECT in Different Murine Tumors

The four cell lines previously tested in vitro, which form tumors with distinct immunological profiles, were also used as tumor models for ECT treatment. Treatment effectiveness was assessed and compared among the four distinct tumor models.

**Figure 3:**
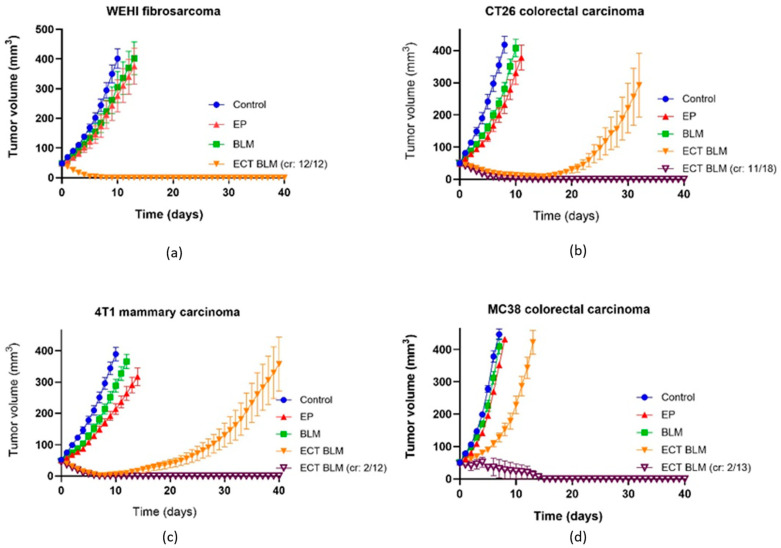
Effectiveness of ECT in murine tumors. Growth curves of WEHI fibrosarcoma (**a**), CT26 colorectal carcinoma (**b**), 4T1 mammary carcinoma (**c**) and MC38 colorectal carcinoma (**d**) are presented as AM ± SE. cr = complete response, mice with cured tumors, number of animals = 12 in all other groups, then ECT. EP = electroporation, BLM = bleomycin, ECT = electrochemotherapy.

ECT was the most effective treatment for WEHI fibrosarcoma with a 100% complete response (CR) rate (Figure 3a), followed by CT26 colorectal carcinoma with a 61% CR rate and a significant GD of 27.5 ± 2.8 days (Figure 3b, Table 1). In contrast, ECT was significantly less effective in 4T1 mammary carcinoma and MC38 colorectal carcinoma, with 17% and 15% CR rates, respectively. Although there was a low CR rate in 4T1 tumors, tumor GD was significantly longer than that in MC38 tumors and not significantly different from that observed in CT26 tumors (Figure 3c,d). Therefore, we can conclude that ECT was the least effective in MC38 when considering both GD and CR rates.

### 3.3. Potentiation of the Antitumor Effectiveness of Combined ECT and Anti-PD-1 Immunotherapy

We further investigated the effectiveness of immunotherapy using a murine anti-PD-1 inhibitor in all four tumor models. The effectiveness of the combined treatment with ECT was also investigated.

**Figure 4:**
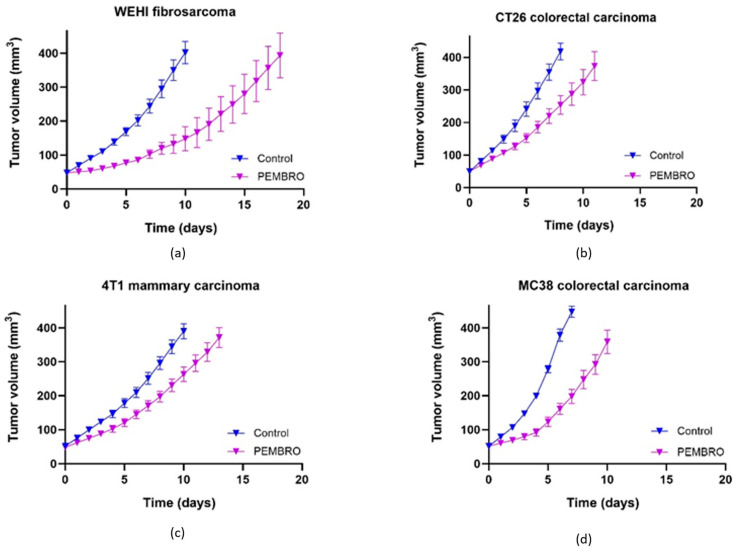
Effectiveness of anti-PD-1 immunotherapy in murine tumors. Growth curves of WEHI fibrosarcoma (**a**), CT26 colorectal carcinoma (**b**), 4T1 mammary carcinoma (**c**) and MC38 colorectal carcinoma (**d**) are presented as AM ± SE. number of animals = 12 in all groups.

Anti-PD-1 immunotherapy alone, similar to ECT, was most effective in WEHI fibrosarcoma (Figure 4a), where GD was significantly longer than that in the other three tumor types (Table 2). In other tumor models, GD after anti-PD-1 treatment was comparable and not significantly different between the tumor models (Figure 4b–d).

**Figure 5:**
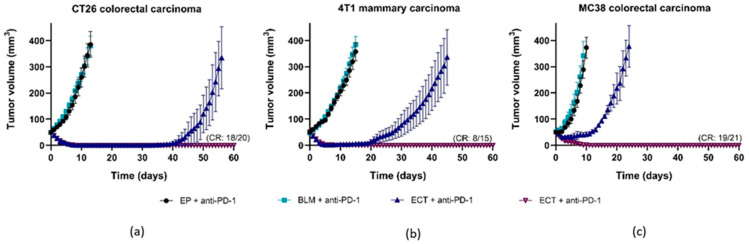
Effectiveness of combined ECT + anti-PD-1 treatment. Growth curves of CT26 colorectal carcinoma (**a**), 4T1 mammary carcinoma (**b**) and MC38 colorectal carcinoma (**c**) are presented as AM ± SE. CR = complete response, mice with cured tumors, number of animals = 15 in all other groups, then ECT + anti-PD-1. ECT = electrochemotherapy, BLM = bleomycin, ECT = electrochemotherapy.

Furthermore, combined ECT and anti-PD-1 therapy was tested. However, as ECT resulted in 100% CR in mice with WEHI fibrosarcoma, the combined treatment was not tested for this tumor type. For the other three tumor types, significant GD was observed following combined ECT + anti-PD-1 treatment (Figure 5a–c, Table 2). In addition to GD, CR rates were also notably high (90%, 53%, and 91% for CT26, 4T1, and MC38 tumors, respectively).

**Figure 6:**
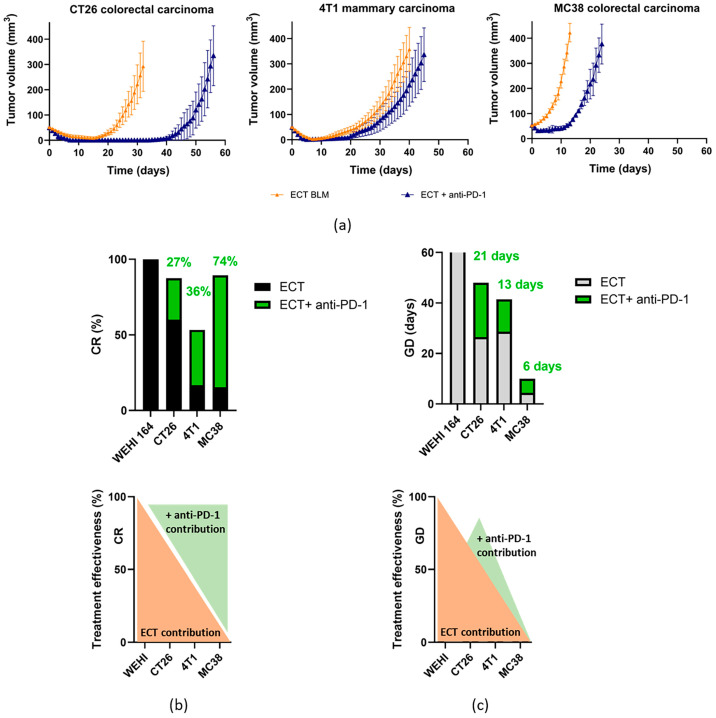
Comparison of treatment effectiveness between ECT and combined ECT + anti-PD-1 treatment. Growth curves (**a**), CR rates (**b**), and GDs (**c**). BLM, bleomycin, ECT, electrochemotherapy.

The effectiveness of ECT alone or in combination with anti-PD-1 therapy was further investigated (Figure 6). In terms of CR rate, WEHI was the most responsive to ECT alone, followed by CT26, whereas 4T1 and MC38 were much less responsive. In terms of CR rate, combined treatment with anti-PD-1 therapy contributed the most to the less responsive MC38 tumors. With respect to GD, the addition of anti-PD-1 therapy further prolonged growth delay, which was proportional to the effect of ECT (Figure 6b).

Taken together, these findings suggest that the addition of anti-PD-1 immunotherapy potentiates the antitumor effectiveness of ECT in mice. Overall, immunotherapy contributed the most to the less responsive ECT treatment.

### 3.4. Immunological Effects

We further examined the immunological characteristics of different tumor types in both untreated controls and treated tumors.

**Figure 7:**
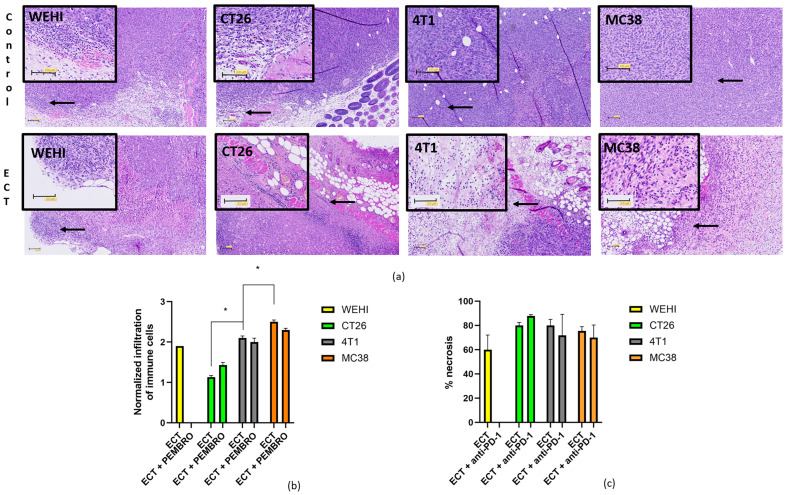
Infiltration of immune cells after treatment. Representative images of immune cell infiltration in control tumors and 3 days after ECT treatment. The black arrows indicate immune cells. The indicated area is zoomed-in in black squares (**a**). The infiltration of immune cells normalized to that of control tumors after ECT or combined ECT + anti-PD-1 treatment. * *p* < 0.05 indicates a significant difference (**b**). Tumor necrosis normalized to that of control tumors 3 days after ECT treatment (**c**). Scale bar = 100 µm. ECT = electrochemotherapy.

First, we determined the infiltration of immune cells into HE-stained tumor sections (Figure 7a,b). In control tumors, infiltration was greater in the WEHI and CT26 tumors than in the 4T1 and MC38 tumors. After ECT and combined treatment, we observed an increased infiltration of immune cells into the tumors. This infiltration was significantly greater after ECT in 4T1 and MC38 tumors, confirming the in situ vaccination effect of ECT. The increase in immune cell infiltration (CT26 < 4T1 < MC38) correlated with the contribution of anti-PD-1 therapy to treatment effectiveness described in the previous section. Tumor necrosis analysis revealed that ECT caused extensive necrosis (Figure 7c), resulting in antigen exposure that subsequently led to immune cell infiltration.

**Figure 8:**
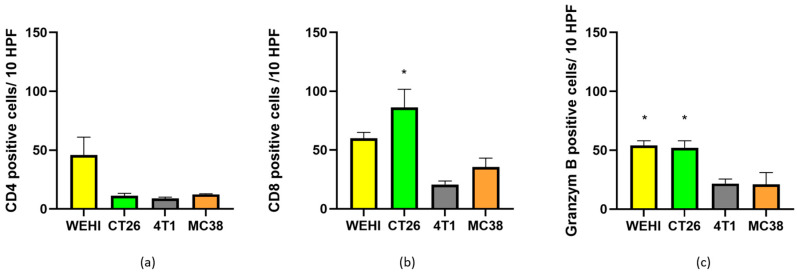
Presence of CD4 (**a**), CD8 (**b**) and granzyme B (**c**) positive cells in nontreated control tumors. * *p* < 0.05 indicates a significant difference compared with the 4T1 tumor model (**b**,**c**).

In addition to the total number of immune cells in tumors, immune cell subpopulations also play a major role in treatment effectiveness. Therefore, we determined the presence of CD4+, CD8+, and granzyme B-positive cells in control tumors (Figure 8). These values served as a baseline for tracking changes in immune cell subpopulations after treatment (Table 3).

Furthermore, we investigated the changes in immune cell subpopulations after ECT or combined ECT and anti-PD-1 treatment. We focused particularly on effector lymphocytes, namely helper (CD4) and cytotoxic (CD8, GrB) lymphocytes, which are the primary targets for anti-PD-1 therapy.

The highest immune-associated score (Table 3) in control tumors was calculated for WEHI and CT26 tumors, which were also more responsive to ECT than the 4T1 and MC38 tumors. The WEHI, which had a 100% CR rate, had the highest calculated immune-associated score after ECT. In the MC38 tumor model, the immune-associated score after ECT was higher than that after 4T1, although 4T1 responded better to ECT. After combined therapy, the highest immune-associated score was calculated for CT26 and MC38, both of which responded with a high complete response rate (app. 90%).

**Figure 9:**
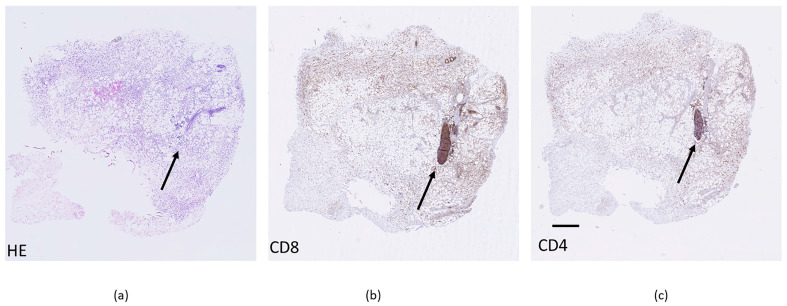
Tertiary lymphoid structures within MC38 tumor with different stains; HE (**a**), CD8+ lymphocytes (**b**) and CD4+ (**c**). Scale bar = 1 µm. Arrows indicating position of tertiary lymphoid structure.

Moreover, newly formed tertiary lymphoid structures have been observed in some tumors after combined ECT and anti-PD-1 immunotherapy. These structures were observed mainly in MC38 tumors (3/4), and rarely in CT26 and 4T1 tumors (1/4). The structures were positive for CD8+ and CD4+ staining, indicating a high density of T lymphocytes, which are primary targets for immunotherapy (Figure 9).

**Figure 10:**
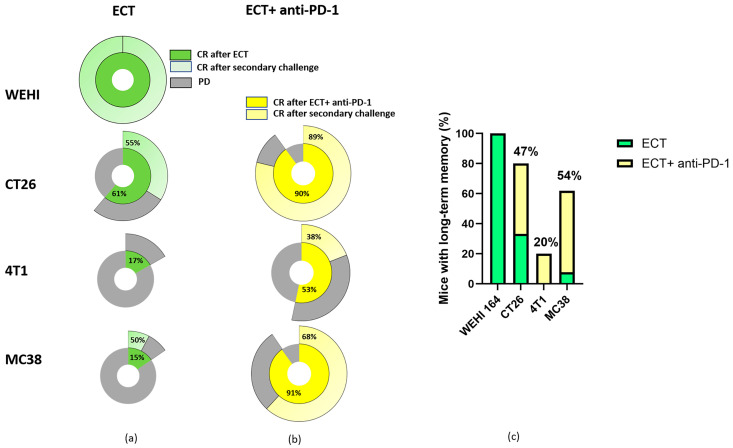
Long-term immune memory of treated mice. Tumor response after primary treatment (inner circle) and after secondary challenge with tumor cells (outer circle) for ECT alone (**a**) or in combination with anti-PD-1 (**b**). Long-term immune memory after ECT (green) or ECT + anti-PD-1 treatment (green + yellow) was calculated for all treated mice. The yellow percentage indicates the contribution of anti-PD-1 treatment in combined ECT + anti-PD-1 therapy (**c**). ECT = electrochemotherapy, PD = progressive disease.

One of the primary aims of immunotherapy is not only to eliminate existing tumor cells but also to stimulate long-term immune memory. Therefore, we investigated how mice that respond completely to ECT or combined ECT + anti-PD-1 therapy respond to a secondary challenge. WEHI fibrosarcoma-bearing mice responded to ECT with a 100% CR rate. All mice also achieved CR after secondary challenge with a subcutaneous injection of WEHI cells, which was very encouraging and indicated that ECT can induce long-term immune memory in mice bearing WEHI tumors (Figure 10a). For the CT26 and MC38 tumor models, only half of the mice that responded with CR to ECT treatment also maintained CR after the secondary challenge, with a higher overall proportion (considering all treated animals) in the CT26 tumor model. In contrast, no long-term immune memory was observed at 4T1 after the ECT treatment, most likely due to immunosuppressive microenvironment of 4T1 tumor (Figure 10a).

When ECT was combined with anti-PD-1 immunotherapy, CR rates further increased compared with those of ECT monotherapy. More importantly, long-term immune memory increased in all tumor types (Figure 10b,c). In the CT26 tumor model, 90% of the mice responded to the combined treatment, and almost all of them (89%) developed long-term immune memory, that is, they remained in CR after the second challenge. Although a 90% CR rate was also observed after combined therapy in MC38 mice, a much lower percentage of mice (68%) developed long-term immune memory. 4T1-bearing mice also benefitted from combined therapy; 53% achieved CR after combined treatment, and 38% developed long-term immune memory (Figure 10b).

When long-term memory was compared after ECT and combined ECT + anti-PD-1 treatment, an increase after combined therapy was observed in all three tumor models (Figure 10c). Compared with ECT alone, the contribution of anti-PD-1 immunotherapy (Figure 10c, yellow bars) to long-term immune memory (Figure 10c, green bars) was greater in the MC38 and CT26 tumor models (54% and 47%, respectively). In 4T1 tumors, only the combination of anti-PD-1 immunotherapy elicited long-term immune memory (20%). Fisher’s exact test demonstrated significantly higher CR rate in the ECT + anti-PD-1 group compared with ECT alone in CT26 (*p* = 0.0076; OR = 0.125; 95% CI 0.02–0.60) and MC38 tumors (*p* = 0.0033; OR = 0.051; 95% CI 0.003–0.47). There was no significant difference CR rate after ECT + anti-PD-1 group compared to ECT alone in 4T1 tumors (*p* = 0.231). Nevertheless, reduced tumor burden after ECT enabling priming, could also play a key factor in immune memory formation.

## 4. Discussion

Several different treatment strategies, including local and systemic strategies, are used in the management of advanced metastatic disease. One such method is ECT, a combination of a cytotoxic drug and electric pulses, which facilitates drug uptake into the targeted tissue [23,24,25,26]. As it induces ICD and facilitates an immune-mediated antitumor response, it has emerged as a potential candidate for combination with immunotherapy, which is currently the standard of care for the treatment of different advanced cancers [27,28,29,30]. Numerous clinical studies have investigated the potential of immunotherapy for the treatment of other types of cancer where it is not yet a standard therapy [31,32]. A recent clinical study combining ECT with the immune checkpoint inhibitor pembrolizumab in patients with advanced melanoma demonstrated promising results in improving the response rates and overall survival [3]. Therefore, in the present nonclinical study, we confirmed the rationale for combining ECT with ICIs by demonstrating the efficacy of the combined therapy in four histologically and immunologically different mouse tumor models and elucidating some of the underlying mechanisms of such combined therapy.

In our previous study, we reported that ECT alone, as well as in combination with immunotherapy (IL-12), is dependent on the immune status of the tumor [1]. ECT combined with peritumoral IL-12 GET in murine cancer models (melanoma, colorectal carcinoma, and breast cancer) enhances local and systemic antitumor effects. This combination was the most effective in treating poorly immunogenic B16F10 melanoma, resulting in increased CR rates and abscopal effects. In more immunogenic tumors, such as CT26 colorectal carcinoma, IL-12 GET did not significantly improve the ECT outcomes. The effectiveness of the combined therapy was inversely proportional to the immunogenicity of the tumor. Therefore, the results suggest that therapy selection depends on the immune status of the tumor. However, we have to acknowledge that other factors such as stromal composition and vascularity can also greatly influence ECT efficacy.

Although direct comparisons of immune cell infiltration and subpopulation analysis of two or three tumor models have already been published, none of the studies compared all four tumor models at the same time. Stringhini et al. reported that WEHI tumors are largely infiltrated by immune cells, similar to CT26 tumors, with minor lymphocyte subpopulation differences [22]. Mosely et al. reported that, in CT26 cells, tumor immune infiltrates are rich in cytotoxic immune cells, whereas 4T1 and MC38 cells are predominantly composed of immunosuppressive cell types: granulocytic myeloid-derived suppressor cells (gMDSCs, >40% of immune infiltrates in 4T1 cells) and monocytic MDSCs (mMDSCs, 47% in MC38 cells). Moreover, compared with other models, CT26 tumors contained the highest number of NK cells [21]. In the present study, we observed similar lymphocyte infiltration in tumors as previously reported and confirmed that the effectiveness of ECT correlates with the immune status of tumors, that is, WEHI and CT26, which represent “hot tumors”, responded better than 4T1 and MC38, which are defined as “cold tumors”. Nevertheless, the intrinsic sensitivity of tumor cells, such as drug uptake and electroporation threshold, could also contribute to good effectiveness.

The effectiveness of ECT alone was the highest in WEHI tumors, followed by CT26, 4T1, and MC38 tumors, which correlated with both the intrinsic tumor cell line sensitivity and the immune status of the tumor. When immunotherapy was added to ECT treatment, the antitumor effect was enhanced in all three tumor types (CT26, 4T1, and MC38), except for WEHI, which had already achieved 100% CR after ECT alone, most likely due to intrinsic tumor immunogenicity. The addition of immunotherapy contributed the most to less responsive tumors after ECT treatment. Similar observations were reported by Ursic et al., in which IL-12 GET was used as immunotherapy combined with ECT [1]. However, we must emphasize that anti-PD1 and IL-12 have different mechanism of immune activation. IL-12 drives Th1 differentiation, strongly enhances NK and CD8^+^ T-cell cytotoxicity, and induces high levels of IFN-γ, whereas PD-1 inhibitors restore function in already primed, chronically stimulated T cells by releasing SHP-2–mediated inhibitory signaling [33,34,35].

Other preclinical studies have indicated that ECT can enhance antigen presentation and T cell infiltration, thereby synergizing with ICIs [1,11]. In the present study, we observed that ECT caused extensive necrosis (>60% of the tumor area). After ECT, tumor antigens are presumably exposed and can attract immune cells to the tumor. Immune cell infiltration after ECT was observed in HE-stained tumor sections from all four tumor types. This observation confirmed that ECT indeed has an in situ vaccination effect, leading to the infiltration of immune cells, including CD4+ and CD8+ lymphocytes, which are primary targets for anti-PD-1 immunotherapy. Moreover, we observed that the increased infiltration compared to that in untreated tumors was the most prominent and significantly different in MC38 tumors, followed by that in 4T1 and CT26 tumors. The increase in infiltrated immune cells correlated with the contribution of immunotherapy to ECT. The limitation of the study is that our analysis measured only the abundance of CD4^+^, CD8^+^, and granzyme B^+^ cells; but functional markers such as CD69, Ki67, or IFN-γ production were not evaluated.

Moreover, after combination therapy in some tumor samples, we observed newly formed tertiary lymphoid structures and organized aggregates of immune cells that formed in non-lymphoid tissues [36]. They were mostly present in MC38 tumors, in which the contribution of immunotherapy was most prominent. Its presence within tumors could have implications for antitumor immunity and clinical prognosis, such as improved patient survival and better responses to immunotherapies in cancers, such as melanoma, NSCLC, and breast and colorectal cancers [37,38]. Our findings suggest that combined therapy may not only elicit an acute antitumor effect, but also facilitate local immune priming, effectively converting immune-excluded “cold” tumors into inflamed, therapy-responsive “hot” tumor phenotypes. However, we have to emphasize that TLS maturity cannot be confirmed since identification was only performed morphologically and by T-cell staining, and markers of mature TLS, such as PNAd or CXCL13, were not evaluated. This study is also lacking functional assays supporting that the formation of TLS implied improved prognosis.

The correlation between increased immune infiltration and tumor regression in less immunogenic tumor models underscores the potential of ECT as an immune adjuvant. The ability of ECT to release tumor antigens and DAMPs may functionally resemble that of in situ vaccination, as supported by the increased CD8+ lymphocyte infiltration and granzyme B activity in treated tumors. Importantly, the observation that anti-PD-1 did not significantly increase immune infiltration beyond that induced by ECT alone suggests that ECT is the primary driver of immune cell recruitment, whereas PD-1 blockade primarily enhances effector functions and memory formation. This was clearly shown in the 4T1 tumor model, where immune memory was induced only after combined treatment. In addition, the proportion of mice with long-term immune memory in the CT26 and MC38 tumor models also increased after the combined therapy.

The inability of mice to form immune memory may be due to the high infiltration of MDSCs into tumors. MDSCs affect immune memory primarily by suppressing T cell responses, which are crucial for long-term immune protection [39]. In our study, the two tumor types with higher MDSC levels in untreated tumors, MC38 and 4T1, presented low or no CR rates after the secondary challenge, demonstrating the inability to induce long-term immune memory after ECT alone. Immune memory develops after combining ECT with anti-PD-1 immunotherapy. Some studies have suggested that immunotherapy can eliminate or mitigate the immunosuppressive functions of MDSCs [40]

The limitation of our study is that it focused solely on effector lymphocytes without accessing specific subpopulations, such as NK cells or immunosuppressive cell subpopulations, such as regulatory T cells (Tregs), tumor-associated macrophages (TAMs), or MDSCs. This restricts our ability to fully understand the immunological landscape and potential mechanisms of immune evasion or regulation.

From a translational perspective, our findings suggest stratifying patients based on their tumor immune status when considering ECT in combination with immunotherapy. While tumors, such as sarcomas, may respond well to ECT alone, less immunogenic tumors may benefit significantly from additional immunotherapy. In this systematic review and meta-analysis, which evaluated the clinical effectiveness of ECT across 44 studies involving 1894 tumors, ECT was more effective in various soft tissue sarcomas than in carcinomas or melanoma tumors. Later, analysis of the International Network for Sharing Practices on Electrochemotherapy (InspECT) database confirmed that the response of sarcomas was better than that of carcinomas or melanomas [4,41].

In clinical settings, melanoma treatment has increasingly focused on combination therapy to overcome resistance to single-agent treatments [27,42,43,44]. The combination of ECT and the anti-PD-1 agent pembrolizumab has shown great potential [3]. In addition to ICIs, other forms of immunotherapy, such as GET of the cytokines IL-2 and IL-12 or chemokine CCL5, could be candidates for combination therapy with ECT. ECT combined with IL-12 GET has already been used in veterinary clinical trials to treat dogs with spontaneous mast cell tumors. Compared with ECT, combined treatment resulted in significantly better local control, longer disease-free intervals, and longer progression-free survival [45]. Our previous findings support the combination of ECT with GET in one session with the same pulse protocol and electrodes, allowing for easier implementation [46].

The results of our current study support the increasing evidence that combining ECT with immunotherapy can transform “cold” tumors, which are resistant to the immune system, into “hot” tumors, which are susceptible to immunotherapy. More specifically, the study demonstrated increased immune-cell infiltration after ECT, suggesting partial immune activation; however, full phenotypic conversion cannot be proven, and additional studies are therefore required to clarify this response.

## 5. Conclusions

This preclinical in vivo study demonstrated that combining ECT with anti-PD-1 therapy significantly enhances antitumor effectiveness, particularly in “cold” murine tumor microenvironment. While ECT alone was highly effective in treating “hot” tumors, the addition of PD-1 blockade improved the complete response rates, immune infiltration, and long-term immune memory in resistant models.

These results exhibit vaccine-like features of ECT, including antigen release and immune recruitment, and the ability of anti-PD-1 therapy to amplify immune activation. These findings support a tailored approach to cancer therapy that combines ECT with immunotherapy based on tumor immune status.

## Figures and Tables

**Table 1:** Treatment response of WEHI, CT26, 4T1 and MC38 tumors to ECT. WEHI tumors were the most responsive, and MC38 tumors were the least responsive.

	WEHI	CT26	4T1	MC38
Immune status	++	++	+/−	+/−
Treatment	GD (days)	GD (days)	GD (days)	GD (days)
	CR (%)	CR (%)	CR (%)	CR (%)
EP	2.7 ± 0.8	2.1 ± 0.5	2.7 ± 0.6	1.2 ± 0.1
	0	0	0	0
BLM	1.7 ± 0.6	0.8 ± 0.2	1.1 ± 0.2	0.5 ± 0.1
	0	0	0	0
ECT	/	**27.5 ± 2.8**	**28.6 ± 2.8**	**4.4 ± 0.6**
	**100**	**61**	**17**	**15**

GD, growth delay; CR, complete response; EP, electroporation; BLM, bleomycin; ECT, electrochemotherapy. The values are presented as AM ± SE; ++ tumors largely infiltrated with cytotoxic immune cells, +/− tumors predominantly infiltrated with myeloid-derived suppressive cells [21,22].

**Table 2:** Treatment response of WEHI, CT26, 4T1 and MC38 tumors to ECT combined with anti-PD-1 immunotherapy.

	WEHI	CT26	4T1	MC38
Treatment	GD (days)	GD (days)	GD (days)	GD (days)
	CR (%)	CR (%)	CR (%)	CR (%)
anti-PD-1	6.6 ± 1.4	1.7 ± 0.7	1.6 ± 0.5	2.6 ± 0.5
	0	0	0	0
EP + anti-PD-1	/	4.5 ± 1.4	2.6 ± 0.5	3.7 ± 0.5
	/	0	0	0
BLM + anti-PD-1	/	2.7 ± 0.9	2.5 ± 0.4	2.6 ± 0.7
	/	0	0	0
ECT + anti-PD-1	/	**48.0 ± 4.4**	**41.4 ± 8.6**	**10.1 ± 0.4**
	/	**90**	**53**	**91**

CR, complete response; EP, electroporation; BLM, bleomycin; ECT, electrochemotherapy. Values are presented as AM ± SE.

**Table 3:** Table presenting the immune-associated scores of four different parameters, namely, CD4+, CD8+, Granzyme B and lymphocyte infiltration, for four different tumors after ECT or ECT + anti-PD-1 treatment.

	Group	CD4+	CD8+	GrB	Infiltration	Sum
WEHI	CTRL	1	1	1	1	4.0
	ECT	1	1	1	2	5.0
	ECT + anti-PD-1	/	/	/	/	/
CT26	CTRL	0.5	1	1	1	3.5
	ECT	1	1	1	1	4.0
	ECT + anti-PD-1	1	0.5	1	1	3.5
4T1	CTRL	0.5	0.25	0.5	0.5	1.75
	ECT	0.5	0.5	0.5	1	2.5
	ECT + anti-PD-1	0.5	1	0.5	1	3
MC38	CTRL	0.5	0.5	0.5	0.5	2
	ECT	1	0.5	0.5	1	3
	ECT + anti-PD-1	1	1	0.5	1	3.5

## Data Availability

All data needed to evaluate the conclusions are presented in the paper. All materials, data, and protocols described in the manuscript will be made available upon request.

## Abbreviations

The following abbreviations are used in this manuscript:

EP	Electroporation
BLM	Bleomycin
CR	Complete response
ECT	Electrochemotherapy
GET	Gene electrotransfer
MDSC	Myeloid derived suppressor cell
PD	Progressive disease
PD-1	Programmed cell death receptor 1
TLS	Thertiary Lymphoid Structures
